# Editorial: Sex and the Suffering Brain - A Call for Sex-Stratified Analyses in Psychiatric Research

**DOI:** 10.3389/fpsyt.2022.849009

**Published:** 2022-03-04

**Authors:** Claudia Barth, Gabriëlla A. M. Blokland, Anita Riecher-Rössler

**Affiliations:** ^1^Department of Psychiatric Research, Diakonhjemmet Hospital, Oslo, Norway; ^2^Norwegian Centre for Mental Disorders Research, Institute of Clinical Medicine, University of Oslo, Oslo, Norway; ^3^Department of Psychiatry and Neuropsychology, School for Mental Health and Neuroscience, Faculty of Health, Medicine and Life Sciences, Maastricht University, Maastricht, Netherlands; ^4^Medical Faculty, University of Basel, Basel, Switzerland

**Keywords:** depression, first episode psychosis, sex differences, chronic schizophrenia, antipsychotics—treatment, sex- and gender-based medicine (SGBM)

In 2005, *Science* published an article about “Sex and the Suffering Brain,” highlighting the need for research into the biological underpinnings of widespread sex differences in the prevalence, symptomatology, and progression of numerous illnesses affecting the brain (1). For instance, while females are about twice as likely as males to develop severe depressive episodes during their lifespans (2, 3), they develop schizophrenic psychoses on average 4–5 years later than males and have a better course of this disorder (4). Yet, despite recent advances in sex-and-gender-based research approaches in both North America and Europe, such as the “Sex-as-a-biological-variable” NIH initiative (5) and the European Commission's “Gendered Innovations” policy (6), the underlying mechanisms for these prominent sex differences are still largely unknown, 16 years after the *Science* article was published.

The study of sex differences has largely been neglected in basic and preclinical research (7, 8). Pre-clinical studies often only included males, or analyzed data from males and females collectively, obscuring differences between the sexes. The resulting sex bias in medicine can have grave consequences for patients' wellbeing and health equity, particularly in the context of women's health (9, 10). Sex-stratified approaches are crucial to studying individual differences in clinical symptoms, treatment responses and outcomes in psychiatric illnesses. By using such an approach, the current Research Topic aimed at shedding light on how sex-specific factors contribute to mental health in females and males.

The Research Topic includes a critical mini-review of the pharmacodynamics of antipsychotics in both sexes (Seeman) as well as three research articles examining sex differences in first-episode psychosis (FEP) (Gjerde et al.), chronic schizophrenia (Yang et al.) and depression (Piani et al.), respectively.

Female sex is a significant risk factor for reporting a severe side effect burden to antipsychotics (11, 12). Women with psychotic disorders show an increased susceptibility to weight gain, diabetes, and cardiovascular risks (13). In her review, Seeman details how antipsychotic drug efficacy and tolerability are influenced by biological sex (e.g., drug absorption, distribution, metabolism, and elimination) and gender-associated social roles (e.g., drug adherence, likelihood to report side effects, lifestyle). She highlights that standard antipsychotic doses are often too high for women, compared to men. This “overdosing” of female patients warrants attention in clinical practice to avoid unnecessary adverse effects (14). For instance, adjusting for weight and hormonal status, as well as avoiding polypharmacy, may help lower the side effect burden in women. However, genetic factors such as drug-metabolizing cytochrome P450 isozymes and chronological age may modulate antipsychotic pharmacodynamics to an even greater degree than biological sex or social gender roles (Seeman). Hence, these factors ought to be considered to improve therapeutic efficacy and clinical outcomes for men and women.

Corroborating Seeman's review, Gjerde et al. studied the nature of antipsychotic-induced metabolic disturbances in a cross-sectional sample of 283 male and 152 female patients with FEP (males: mean age 26.7 ± 7.6 years, females: 27.4 ± 9.0). Specifically, the authors investigated the relationship between body mass index (BMI), serum lipid measures [total cholesterol, high/low-density lipoproteins (HDL-C/LDL-C), and triglycerides] and psychotic as well as depressive symptoms. Relative to female patients, male patients had a higher mean BMI, higher LDL-C and triglyceride levels, and lower HDL-C levels. In females with FEP, the authors demonstrate a significant negative association between HDL-C levels and negative symptoms, and a significant positive association between BMI and depressive symptoms. While these findings suggest a link between favorable lipid profiles and better therapeutic response, they also highlight the importance of weight management early in the disease progression to subsequently improve adherence and treatment outcomes (15).

Yang et al. investigated metabolic disturbances in 136 females and 196 males with chronic schizophrenia (males: mean age 44.9 ± 11.9 years, females: 45.9 ± 11.6) by zeroing in on insulin resistance, a risk factor for diabetes and for cardiovascular disease (Yang et al.). Levels of insulin resistance, assessed using the homeostasis model assessment of insulin resistance (HOMA-IR), were significantly higher in female patients compared to male patients. This sex difference in HOMA-IR among patients with chronic schizophrenia is a novel finding, and may be linked to changes in sex hormone levels. Women's insulin sensitivity decreases during the transition to menopause, with the cessation of ovarian function increasing women's risk for metabolic disturbances. The work of Yang et al. suggests that this risk may be exacerbated in women with chronic schizophrenia. Hence, reproductive stage and metabolic factors may be routinely monitored in clinical practice to optimize treatment regimens for female patients.

Taken together, the work by Gjerde et al., Yang et al., and Seeman suggests widespread sex and gender differences in antipsychotic treatment response and likely-related metabolic disturbances. Their work highlights the need for more multifactorial and longitudinal research investigating potentially differing causal pathways to illness presentation and treatment response in men and women. Not doing so undermines scientific validity, and results in a failure to deliver sex- and gender-sensitive treatments (9) as part of precision psychiatry.

Besides sex differences in antipsychotic pharmacodynamics and metabolic factors, emerging evidence also suggests sex-specific brain correlates in mental disorders. Piani et al. contributed to this field of research with a pilot study, investigating sex differences in brain morphology and brain function during a Go/No-Go paradigm in adult-onset depression (Piani et al.). The authors found sex-specific effects in brain regions involved in attention processing and in the brain default mode network. Female patients presented with lower gray matter volume and less task-based brain activation than their male counterparts—potentially supporting sex differences in disease presentation. However, as the sample size is small, firm conclusions cannot be drawn and more research is warranted.

In summary, the current Research Topic provides a window into how individual differences in clinical symptoms, treatment responses and outcomes may manifest in mental disorders across both sexes. Understanding how sex and gender contribute to psychiatric illness across the lifespan provides the opportunity to more precisely target interventions and improve clinical outcomes for both sexes, subsequently reducing health care costs and benefiting society on an individual and global level.

## Author Contributions

CB drafted and finalized the editorial. GB and AR-R critically revised the first draft and approved the final editorial. All authors contributed to the article and approved the submitted version.

## Conflict of Interest

The authors declare that the research was conducted in the absence of any commercial or financial relationships that could be construed as a potential conflict of interest.

## Publisher's Note

All claims expressed in this article are solely those of the authors and do not necessarily represent those of their affiliated organizations, or those of the publisher, the editors and the reviewers. Any product that may be evaluated in this article, or claim that may be made by its manufacturer, is not guaranteed or endorsed by the publisher.
